# Successful Operation on Harelip

**Published:** 1867-12

**Authors:** Arthur B. Stout

**Affiliations:** San Francisco.


### SELECTED ARTICLES.

### ARTICLE VII.

### *Successful Operation on Harelip.*

By Arthur B. Stout, M.D., of San Francisco.

The result, was obtained by three distinct operations. The first operation was performed at the age of eight months; the second operation at the age of two years and nine months; and the third at three years of age, in March, 1867.

The utmost care was taken that the child should be in fine health before the operation. Children born in the family previous to the present one had no deformity, and an infant born since has a perfect lip. The father has a slight imperfection of the right ala of the nose—the effect of an accidental injury. Whether or not the harelip can be referable to this circumstance can only be conjecture.

As, however, the mother made inquiries, and expressed apprehension in reference to future progeny, I determined to forestall, if possible, any such event by commencing the operation at the earliest date. To allay mental misgivings and apprehensions; to remove the constantly recurring presence of the deformity from the sight of the parent, and demonstrate to doubting eyes that a defect, before which even death seemed a more desirable alternative, could be transformed into an endurable condition, was the inducement to hasten the operation to the earliest period given. It is true the books generally recommend postponing such operations until the lip obtains more ample developement; but to my mind the reasons above mentioned should have the precedence in authority.

By observing carefully the mouth of the infant, it was seen that two splits in the lip ascended high to the ala of the nose. The central portion of lip was very short, and was pressed forward by a bone which projected behind it, and to which it adhered by a large base. This bone was a true intermaxillary bone; but instead of standing vertically between the right and left intermaxillaries, it protruded horizontally, forcing the central lip before it. There was no division in the palatine bones nor in the soft palate. Hence it would be difficult to ascribe this harelip to an arrest of developement on either side of the child's body.

The first operation was the excision of the median bone. It was done by dissecting the soft parts from the bone as closely as possible. When the bone was thus exposed it was easy with cutting forceps to remove it by two lateral cuts. A small opening was made in the palatine bone which, however, soon closed and gave no inconvenience. After two years and nine months the second operation was performed, A thin slip of cork was placed under the whole upper lip, which served the double purpose to prevent the flow of blood into the mouth and form a firm basis to cut upon. Over this cork the middle lip was drawn down with force enough to stretch it, and was so pinned fast; the corresponding edge of the lateral lip was then drawn near to it, and pinned to the cork. With a sharp bistoury the edges were cut clean, and immediately the suture pins were introduced, three in number. The cork pins were then removed and the suture thread applied.

The third operation, performed nearly nine months after, was nearly a repetition of the same proceeding. The union of the middle lip to the right lateral lip was done under considerable difficulty, the two edges being of very different length. The admirable suture pins made with little silver cylinders, armed with moveable steel trocar points were used, and proved much better than insect pins. Chloroform was used in each operation, and the closest attention given to the frequent renewal of adhesive straps, both before and after the removal of the pins. In consequence of the strength and opposition of the child, it was necessary to give chloroform to renew the first dressings, and bind him firmly to the table. Too much care cannot be taken with these precautions, as one untimely jerk of the child may frustrate the whole work.—*Pacific Med. and Sur. Journal.*

